# Oldie but Goodie: Is Technetium-99m Still a Treasure Trove of Innovation for Medicine? A Patents Analysis (2000–2022)

**DOI:** 10.1021/acs.jmedchem.3c00148

**Published:** 2023-04-03

**Authors:** Mattia Riondato, Dorotea Rigamonti, Petra Martini, Corrado Cittanti, Alessandra Boschi, Luca Urso, Licia Uccelli

**Affiliations:** # IRCCS San Martino University Hospital, Largo R. Benzi, 10, 16132 Genova, Italy; ‡ European and Italian Patent Attorney, Marchi & Partners S.r.l., Via V. Pisani 13, 20124 Milan, Italy; ⊥ Department of Environmental and Prevention Sciences, University of Ferrara, Via L. Borsari, 46, 44121 Ferrara, Italy; † Department of Translational Medicine, University of Ferrara, Via Fossato di Mortara, 70 c/o viale Eliporto, 44121 Ferrara, Italy; § Department of Chemical, Pharmaceutical and Agricultural Sciences, University of Ferrara, Via L. Borsari, 46, 44121 Ferrara, Italy

## Abstract

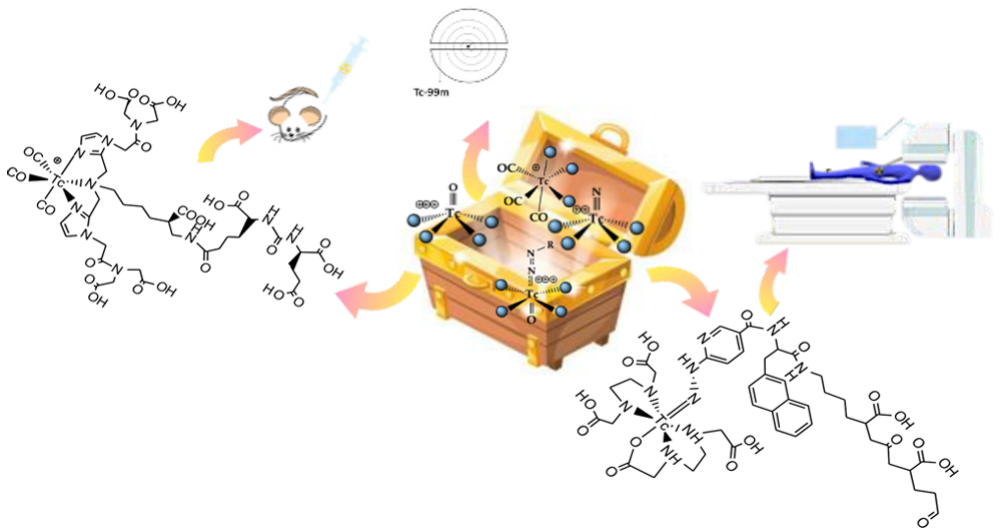

Technetium-99m is the workhorse of diagnostic nuclear medicine. The aim of the work is to analyze the technetium-99m patents since 2000 to photograph its innovation. QUESTEL’s ORBIT Intelligence system was used for the collection of technetium inventions disclosed in patents and patent applications in more than 96 countries in the period 2000–2022; 2768 patent documents were analyzed. Patent counting and analysis have shown that SPECT imaging using technetium-99m radiopharmaceuticals is still robust. The introduction of new technetium-99m radiopharmaceuticals into clinical routine goes beyond successful trials. In eastern economies, such as China and other emerging markets, patent applications are on the rise, while those in developed western countries are stagnating, with some exceptions for the United States. But despite the difficulties, academic and industrial research on these tracers remains essential for the development of nuclear medicine.

## Introduction

Nuclear Medicine is undergoing an unparalleled expansive phase in the field of both positron emission tomography (PET) and single photon emission computed tomography (SPECT).^1^ This is due to the development and diffusion of more efficient instruments, such as hybrid PET/CT and SPECT/CT imaging scanners and, above all, to the introduction of new radiopharmaceuticals.

Novel therapeutic agents are also available in clinics, and the high potential for the application of combined diagnostic and therapeutic agents, so far called the “radiotheragnostic approach”, has especially attracted the attention of large pharmaceutical companies, which are traditionally hesitant to attempt new challenges in this drug market.^2,3^

The main drivers have been the increasing prevalence of the geriatric population and the correlated chronic diseases. Among these pathologies, the rising expenditure in oncology boosted the growth of the diagnostic market, owing to the need for new imaging agents and treatment options for more common, as well as for rare cancers. Finally, the rising awareness of the value of radiopharmaceutical applications in disease treatment also played a key role, especially for clinicians who were diffident about the use of nuclear procedures.^4^

In this context, the perspective of SPECT is also rapidly changing, thanks to advances in solid-state detector technology that involves rapid imaging and high spatial resolution in addition to high sensitivity. The consequence is an evolutionary impulse that feeds the rediscovery of technetium-99m, a still unique radionuclide within the scenario of nuclear medical diagnostics for its ideal nuclear properties and the easy preparation of its radiopharmaceuticals.^5^ But let us take a step back. The discovery of “Tecneto”, from the Greek Τεκνητóς = artificial, is to be attributed to C. Perrier and E. Segrè who, in Palermo in 1937, identified it in the form of technetium-95 and technetium-97 in some samples of molybdenum irradiated with deuterons in the cyclotron of the University of California at Berkeley.^6,7^ For a long time, technetium was considered just an “exotic” artificial element and not too much attention was paid to it. This changed when Brookhaven National Laboratory discovered the ^99^Mo/^99m^Tc generator and the very favorable properties of the metastable nuclide ^99m^Tc for Nuclear Medicine purposes,^8^ so that clinical examinations based on ^99m^Tc still outperform those of PET agents.^9−13^

In addition, the production of technetium-99m is easy, very fast, and inexpensive; moreover, its excellent physicochemical properties, such as a *t*_1/2_ of 6.022 h, a pure γ emission of energy equal to 142 keV, and scarce attenuation by tissues which make it ideal for detection by γ camera allow for a low dose/patient administration in combination with good external detection for image generation. Last but not least, technetium-99m has a very versatile chemistry: its wide range of oxidation states gives the radioisotope an almost unlimited possibility of coordination and consequently the ability to give rise to a wide variety of radiopharmaceuticals.^5^

^99m^Tc-radiopharmaceuticals have been supporting diagnostic Nuclear Medicine activities for decades for functional imaging of the brain, skeleton, kidneys, hepatobiliary tract, and myocardium and for the evaluation of other diseases.^14^ Most of the currently used single photon emitting radiopharmaceuticals were introduced into clinical practice more than 30 years ago, when the medical advances and needs, as well as industry and pharmaceutical regulatory frameworks, were deeply different.

After a long time, this class of diagnostics is still extensively used due to established procedures in all Nuclear Medicine worldwide, either obtained from external commercial suppliers or, as in the case of ^99m^Tc-agents, prepared in-loco for immediate use after radiolabeling of a lyophilized kit, in few minutes, with a physiological solution containing ^99m^Tc-pertechnetate. Technetium-99m is easily available from relatively inexpensive generators, which are distributed to Nuclear Medicine units, supporting more than 70% of the total nuclear imaging procedures with a potential impact on direct patient care.^15^

However, international legislation changed significantly over the years, strengthening the pharmaceutical regulatory framework. According to this evolution, radiopharmaceuticals that are commercially distributed must have a marketing authorization (MA) released by Competent Authorities. Technetium-99m is no exception to this, considering within the pharmaceutical frame not only the diagnostic agents but also two other important categories such as generators and radiopharmaceutical preparation kits (also called “cold” lyophilized kits).^16^

Radiopharmaceutical companies have faced several important threats during the last decades, with a substantial impact on the evolution of Nuclear Medicine, conditioned by the limited market revenue compared with the traditional pharmaceutical industry.^17^ The general implementation of both pharmaceutical and nuclear regulatory requirements has indeed increased the overall cost of radiopharmaceutical manufacturing. Furthermore, technetium-99m availability has suffered for its fragile supply chain, which combines a mix of governmental and commercial interests. A major shortage in 2009–2010, due to a malfunction of aged reactor production facilities, opened officially the crisis and the roundtable over the potential role of ^99m^Tc-agents in the future.^18^

Fortunately, tough times provide also new opportunities for innovation. The installed base of SPECT cameras is still far superior to PET in terms of number and dissemination worldwide; thus this market remains rewarding for potential investors.^19^ Moreover, technetium-99m is still attractive as, due to its large availability, it might accelerate the introduction of new radiotherapeutics into the clinical practice, where imaging and therapeutic intervention are closely linked.^20,21^

For these reasons, alternative approaches for ^99m^Tc production have been developed focusing on a novel sustainable availability of this radioisotope, able to compete with the growing market need.^22−26^ At the same time, new labeling methods based on ^99m^Tc never stopped growing, indicating that research in the field is still alive.^27^ Not least, molecular imaging, as other fields in medicine, has gained benefit from the identification of new targets with the concomitant extended repertoire of tools and technologies, developing radiotracers with high specificity and thus moving toward the modern concept of “personalized medicine”.^28,29^

During the last 60 years, Nuclear Medicine has undergone many changes, but the discipline demonstrated to evolve rapidly following the considerable advances in molecular cell biology. New potential drug targets, as well as biomarkers, have become available from genomics and proteomics, providing a new chemist’s workbench for the design of the probe chemical structures. However, PET seemed to better take advantage of this opportunity and the recent achievements in the use of radiopharmaceuticals labeled with positron emitters hardly affected the field of SPECT, raising the question about the role of ^99m^Tc imaging agents.^30,31^

Can we identify and figure out the trends in the development of ^99m^Tc-radiopharmaceuticals, the workhorse of diagnostic Nuclear Medicine?

We are trying to answer these questions by taking a picture of the innovation in this field by outlining the Nuclear Medicine landscape and analyzing the patent production correlated to technetium-99m since 2000, with the intention to devise a simplified method that might be applied also to other radioisotopes. Innovation is one of the main drivers of productivity performance and the promotion of its culture is of utmost importance for the development of all disciplines.^32^ Inventions in new technologies, from both academia and industry, produce publications and patents. Scientific papers might be useful to assess the major emerging areas, but this does not necessarily reflect innovation and industrial interest.^33,34^ On the other hand, patent documents, especially with the increasing availability through digital formats, represent a rich source of technical and business information, wherein industries are prone to describe the results of their research in patent applications, with the aim of protecting them rather than disclosing them in a scientific paper and, of course, with the ambition to access to the largest possible market. In support of this thought, we report a recent publication which revealed that 80% of technology publications link forward to a patent and only 61% of patents refer to a publication.^35^

This is the first global study that evaluates innovation in the ^99m^Tc-radiopharmaceutical field by using patents. The main goal of the study was to objectively identify key areas of development by examining patent data from the past 20 years. We used this data to quantify, evaluate, and highlight innovation trends within individual ^99m^Tc radiopharmaceutical development clusters and disseminate ^99m^Tc market predictions for the coming years.

## Results and Discussion

The evaluation of patent publication activity is a reliable tool to describe the state of innovation in a technology field. The rate of innovation is one of the main indicators to select attractive industrial sectors and for the evaluation of the productivity performance of a specific field. Significant differences can be observed from one technological domain to another, but in any case, the assessment of novelties has demonstrated to predictively drive technological changes and raise funds from investors.

Several studies based on patent data have been proposed in the literature by many authors from 1968 to the present,^36−38^ investigating the relationship between patent counts and innovation performances. The assessments are mostly related only to a specific patent feature, such as the number of patents issued by a company or the number of patents.^39,40^

Major drawbacks on patent counts have also been described. For instance, many patented inventions are not followed by an industrial application or market introduction, while others are not patentable or hardly highlighted because of different regulations across the countries.^41^ However, besides these significant limitations, the patent count is still considered a valuable and simple measure for innovation, correlating the number of patents as direct R&D output.

This work aimed to assess if patent metrics is a valuable tool for identifying and measuring the emergent technetium-99m technological innovations in the field of Nuclear Medicine. The method of analyzing healthcare technology that has been applied was inspired by previous publications, both in the field of radiopharmacy and in other medical disciplines, in order to validate and quantitatively–qualitatively evaluate the disclosed inventions as a marker for innovation in healthcare.^26,42,43^ Furthermore, in the applied methodology, other relevant features (such as the patent owners, firms, and country) were qualitatively analyzed for each patent by the authors, to better appreciate the value the market potentially gives to technetium-99m technologies.

### ^99m^Tc-Patent Document Extraction, Selection, and Evaluation

A first-row data set containing filed patent documents was collected using ORBIT Intelligence. The selected time frame for the extraction, January 2000 to February 2022, is congruent with patent life, which is regularly no longer than 20 years from the date the patent application was filed. There are exceptions to this rule, mainly related to the Supplementary Protection Certificates, allowable for pharmaceutical protection products that have been authorized by regulatory authorities and not considered for the purpose of the current analysis.

We identified the keywords “+99MTC+” or “TECHNETIUM” as the search query that best retrieves technetium-related patents, yielding the first cluster of 2768 patent documents. Each item was referred to a single patent family, wherein a patent family is a group of patents having the same priority, filed in various countries to protect a single invention (i.e., the same invention disclosed by a common inventor or investors and patented in more than one country).

The patent cluster was classified according to technological fields. The most important and harmonized grouping specific to patents is the International Patent Classification (IPC), the most used patent classification system worldwide, established by the Strasbourg agreement in 1971.

**Figure 1:**
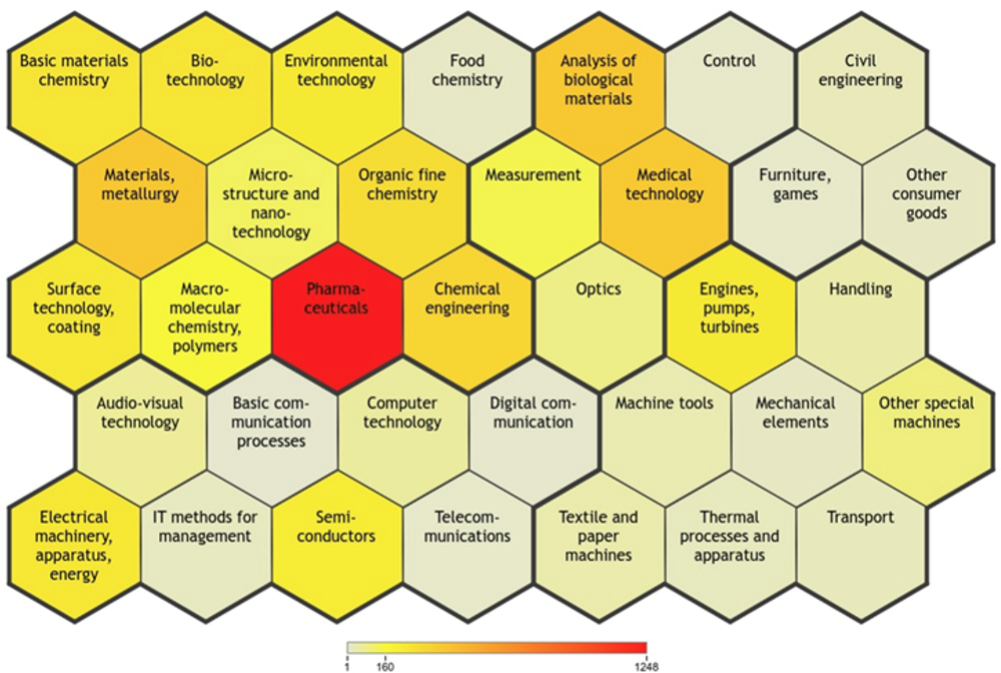
Group of 35 technology fields based on the IPC codes (cluster of 2768 patent documents). Colors represent the patent number in the field, in particular: red = most numerous and gray = least numerous.

The data set was grouped into 35 technology fields based on the IPC codes, as depicted in Figure 1.

Examining the graphical representation, the most represented technology domains belong to *pharmaceutical* fields, followed by *analysis of biological materials*, and *medical technologies*, as reasonably expected. However, transition metals have a large variety of industrial applications, and technetium displays interesting properties for the development of inventions in the fields of new *metallurgy materials* and *chemical engineering*. In line with these considerations, several IPC classes were found to be distant from the scope of the present analysis. Before the definitive exclusion, the authors sampled the patents distributed in the various ICP classes to be eliminated from the selection: *civil engineering*, *basic communication processes*, *computer technology*, *digital communication*, *IT methods for management*, *semiconductor*, and finally *transport*. The 2768 items were, therefore, processed again excluding ICP classes not pertinent to the use of technetium-99m for Nuclear Medicine applications, thus removing a total of 545 patents and reducing the number of the overall patent documents.

This new data set, named “First Dataset” (*n* = 2223), underwent a two-step cleaning process using specific keyword queries in order to verify the patent content.

First, the documents comprising the keywords “+99MTC+” or “TECHNETIUM” in the independent claims only were filtered. This extraction was performed to exclude documents without any correlation with technetium, but in which the same was simply referred to. Having such keywords in an independent claim was indicative of the fact that the patent/patent application specifically focused on the technology of interest. In the second cleaning step, the residual cluster was further refined extracting the documents comprising the term “RADIO+”, thus restricting the data set of patents/patent applications to 610 items (“Intermediate Dataset”). The use of the “+” symbol after RADIO allowed a more accurate selection of the pertinent papers, including the selection by means of appropriate keywords, such as RADIOpharmaceutical, RADIOtracer, or RADIOdiagnostic, for instance.

Using this strategy based on search queries a relevant number of entries (*n* = 1613) was removed, due to a lack of evidence for the presence of any reference to nuclear imaging. Despite the overall exclusion being likely higher than predicted, a deep cleaning of the data was expected because, although technetium owes its popularity to its metastable nuclear isomer technetium-99m (^99m^Tc), technetium-99 (^99^Tc) also occurs in traces in the earth’s crust and as nuclear reactor waste. The latter isotope exhibits outstanding physical–chemical characteristics, such as anticorrosive and superconductor properties, thus being attractive for engineering interests.^44^ For all these reasons most of the removed patent documents were associated with sewage treatment processes, mainly the removal of metal impurities, or novel compositions of enhanced multimetal catalysts for several processes in manufacturing, pharmaceutical, and petrochemical industries.

In any case, considering the number of excluded patents, the authors decided for checking every single document, verifying the titles, abstracts, and for a few also the contents, one by one, corroborating their irrelevance to the “Intermediate Dataset” (*n* = 610). Some inventions disclosed innovative alloy materials for specific destinations, including for medical scope, however far from molecular imaging application. Curiously, other documents were found hardly understandable, mostly because of language (Orbit Intelligence should allow the consultation of the English version of patent documents) or missing any reference to technetium.

“Intermediate Dataset” contained nearly 25% of the starting patents/patent documents and represented the more specific cluster regarding the use of technetium-99m for nuclear medicine applications. However, this selection is inclusive of all the potential legal status of patents: valid and in force (granted), expired, lapsed due to nonpayment of fees or deemed invalid through successful opposition. Therefore, the software-based analysis was then extended to a further level to select the patent documents that were considered “alive”, thus excluding the cluster of documents not currently granted (*n* = 346).

A total of 221 items, grouped and named “Final Dataset”, remained after the completion of the cleaning process performed by the authors (*n* = 43), containing the “alive” patent documents with specific reference only to the radiopharmaceutical field.

**Figure 2:**
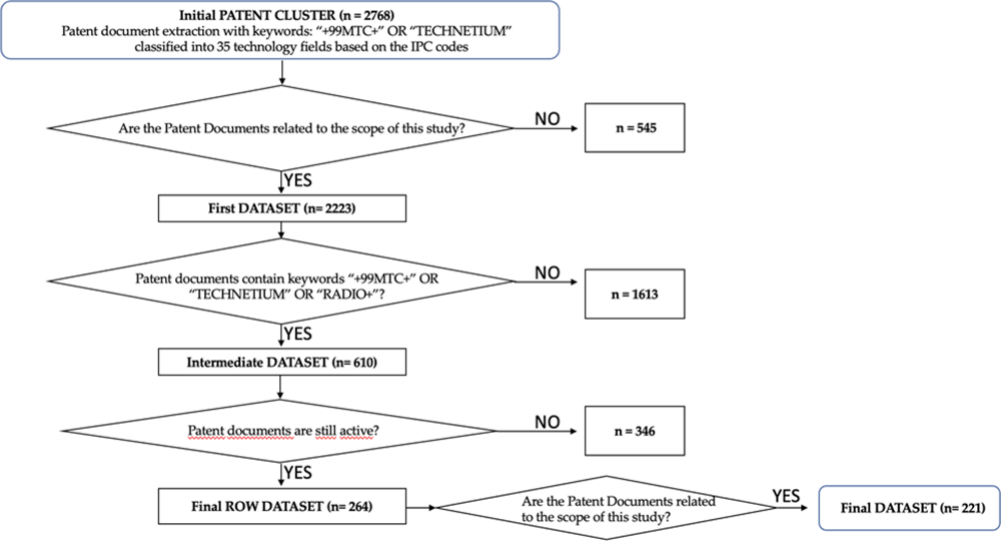
Flowchart for data cleaning operation after first ^99m^Tc-patent document extraction using ORBIT Intelligence and author’s inspection.

A schematic representation of the progression leading to the final selection is reported in Figure 2.

Results and further considerations on the software-based analysis both on the “Intermediate Dataset” and “Final Dataset” are later reported. Indeed, the relatively low number of “alive” patents permitted the authors to perform manually a thorough analysis of the contents, highlighting major areas of innovation and related prominent examples of filed patents.

### “Intermediate Dataset”: Patent Activities by Top Players

**Figure 3:**
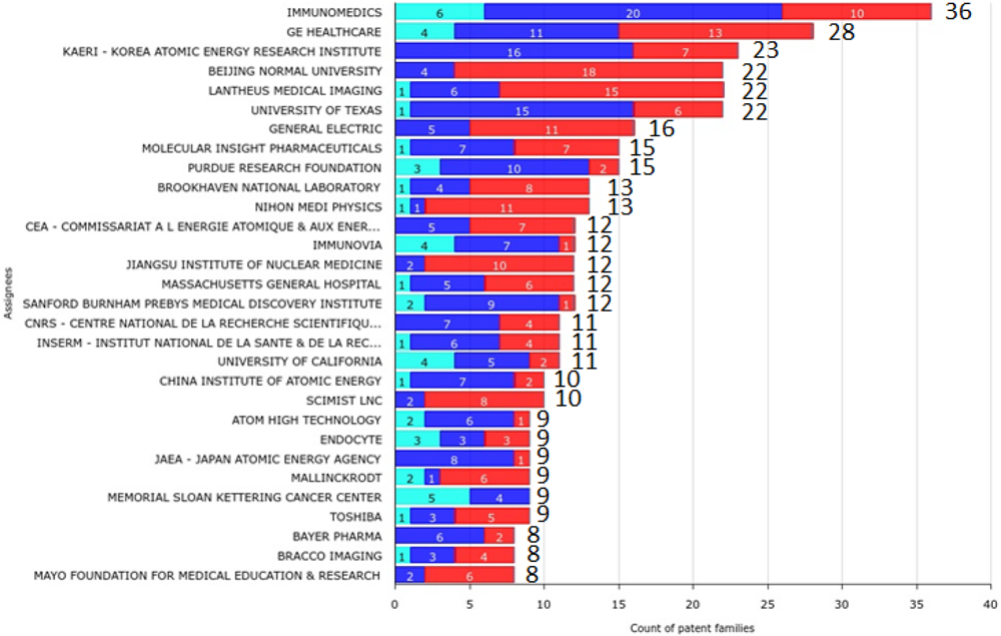
Graph illustrating the 30-top applicants and their related total number of applications in 2000–2022 according to their legal status (light blue, pending; blue, granted; red, dead), adapted from the Questel’s IP Business Intelligence application (Orbit Intelligence).

The Intermediate Data set can be considered the most representative pool of data able to describe the research patent activity in the time frame 2000–2022, including the filed patents per company, research institution, or hospital and their status at the time of this study. The chart in Figure 3 shows the global size of each applicants’ portfolio, divided into pending, granted, and dead patents. Indeed, this graphical representation makes the idea of the dynamics of inventiveness for each active player.

The applicants owning the largest portfolio were Immunomedics and GE Healthcare, from the country where the technetium-99m technology was invented more than 60 years ago at the U.S. Department of Energy’s (DOE) Brookhaven National Laboratory. Indeed, half of the 30 top player companies/institutions have their operational headquarters in the USA, clearly indicating its leader position in this specific field.

The two top applicants are also descriptive of the typical companies interested in this market: the first, Immunomedics (US), is a pure biotechnology company focused on the development of antibody–drug conjugates for the treatment or diagnoses of cancer (Gilead Sciences), while the second is a division of a big corporation providing global medical technologies, including pharmaceutical diagnostics, imaging devices, and digital solutions for patient care.

Other notable patent applicants are present in the major eastern economies, such as China, Korea, Japan, and India, and in the European Union countries traditionally front-runners in the development of diagnostic agents (France, Germany, and Sweden). Among the top players, pharmaceutical companies and research institutions (foundations, hospitals, or universities) are equally represented, an indication of the cross-interest in technetium-99m technologies by both emerging and established companies, mainly driven by the need to monetize their innovation, and research at the basic level of academic institutions, for instance. It is important to keep in mind that universities or other research institutions play a fundamental role in establishing an innovation ecosystem, incubating knowledge-based start-ups, and driving the need for new inventions. Last but not least patents might help them to benefit from additional revenues and to improve their ranking.

**Figure 4:**
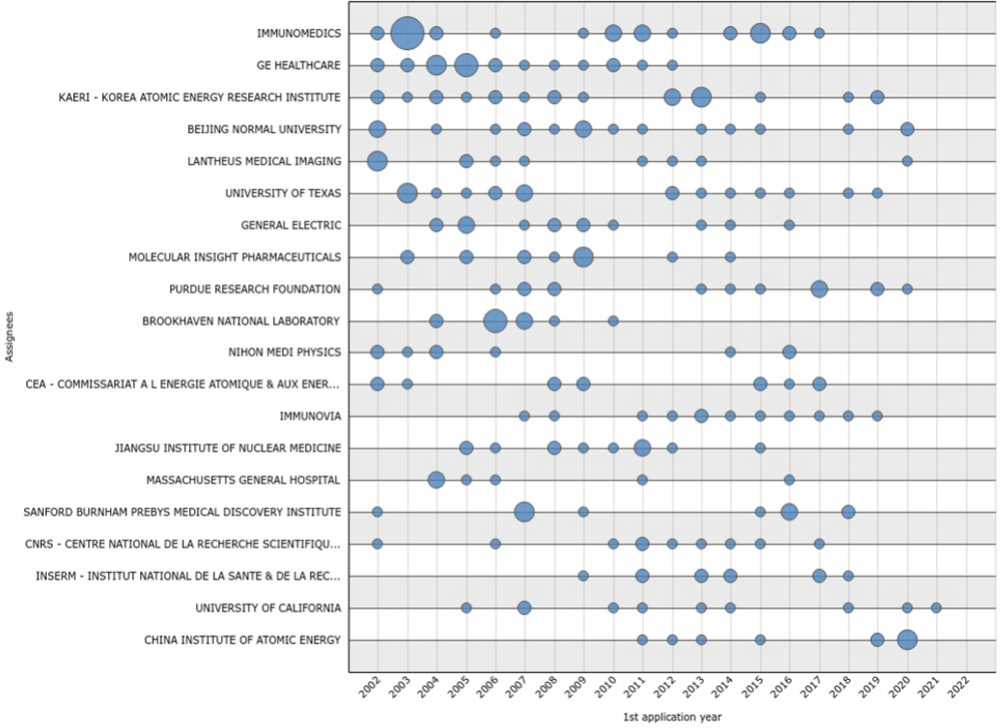
Graph illustrating the evolution of application over time by the 20 top applicants, created on Questel’s IP Business Intelligence application (Orbit Intelligence)

The global analysis of this data set, as mentioned before, represents a valuable indicator of the innovation activities in technetium-99m for nuclear medicine applications over the last two decades. Patents can undergo many status changes, and of course, applicants can also modify their patent strategy or simply they can decide to be no longer involved in this specific field. Figure 4 illustrates the evolution of applications over time by applicant highlighting the investment trend for key players. Different profiles can be observed, depending on the filing strategy implemented by the applicant.

The growing light-blue circle indicates that the applicants are in the phase of construction of their portfolios during the analyzed time frame, as depicted in the graph for Immunomedics and GE Healthcare in 2002–2005, or more recently after 2018 for some eastern institutions, albeit with less intensity, such as China Institute of Atomic Energy, Korea Atomic Energy Research Institute, and Beijing Normal University.

The constant flow of patent applications is generally explained by a large R&D budget availability, able to support both their submission and maintenance. In Figure 4, a stabilization of the number of filings was observed for the majority of the top player applicants during the period 2002–2009 and 2012–2016. The temporary decline, observable at a first glance, in 2010–2011 might be correlated to a contraction of confidence in the future of ^99m^Tc in the diagnostic market determined by the molybdenum-99 crisis.^18^

Lastly, a decline in the number of patents filed is symptomatic of a substantial decline in R&D interest or a reduced intellectual property budget. The case of the top players GE and Immunomedics is particularly evident, missing new applications, respectively, since 2012 and 2018. This evidence does not necessarily mean that these leader companies are not interested anymore in the diagnostic field of nuclear medicine but rather that the marketing strategy changed over the last years for some reasons. The new investments are more likely correlated to radiopharmaceuticals with other radioisotopes, mostly for PET or therapeutic applications. This hypothesis is more consistent for GE, which is not launching new ^99m^Tc-radiopharmaceuticals, substantially maintaining a consolidated market, and shifting the introduction of novelties to fluorine-18 labeled compounds. On the other hand, Immunomedics benefitted for a long time of the special focus nuclear medicine industry for the developments in antibody conjugate technologies, which led to the launch of antibody-targeted radiotherapeutics in the early 2000s (^90^Y-ibritumomab tiuxetan (Zevalin, Spectrum Pharmaceuticals, Inc.) and ^131^I-tositumomab (Bexxar; GlaxoSmithKline)). This approach, however, has historically faced several constraints mainly due to some clinical and manufacturing factors (appropriated patient selection, administration correlated problems, the complexity of production and supply chain) raising the need for switching the research to the development of small molecules and named targeting ligands, exhibiting more favorable properties for use in diagnostic and therapeutic application.^1^ Second, during the past decade, several radioisotopes with better-suited characteristics for labeling longer circulating antibodies have been made available, like zirconium-89 and copper-64. Last but not least, Immunomedics was recently acquired by Gilead in 2020, and this might have an impact on the overall company decision in terms of long-term strategy.

It is not easy to extract univocal conclusions over the representation reported in Figure 4; however, it mirrors a certain degree of change in investments by the traditional players belonging to the consolidated western market. The new main actors for the technetium-99m technological innovation scenario seem to return close to academic and research institutions, particularly government nuclear institutions, with more emphasis in eastern countries. Pharmaceutical companies are not leaving the business; nevertheless, the major players are leaving the place to specialized diagnostic companies (Immunovia, Lanteus Medical Imaging, Molecular Insight Pharmaceuticals, Nihon Medi Physics) and other dynamic small enterprises.

**Figure 5:**
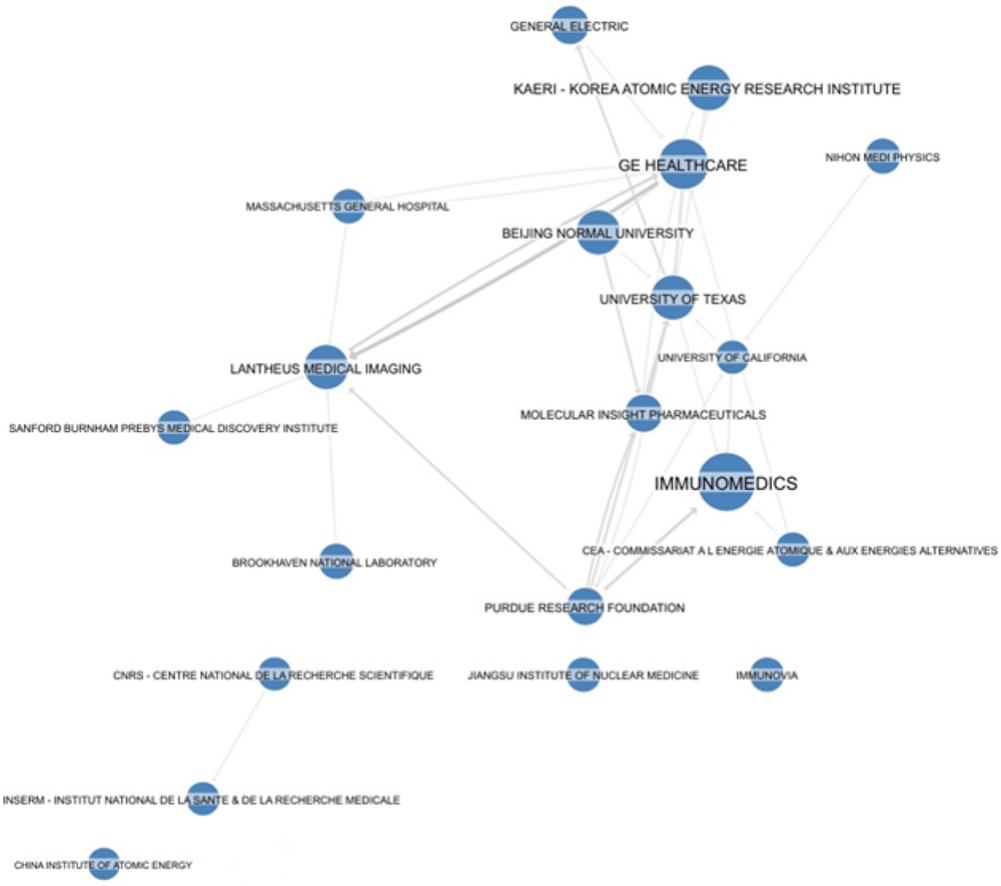
Graph illustrating citations between the 20 top applicants, created on Questel’s IP Business Intelligence application (Orbit Intelligence).

As last presented, analyses of the Intermediate Data set a search to look for cooperation; just a few patent documents are coassigned, as an indication of almost no interaction among players. The interassignee citations are depicted in Figure 5.

Typically, a portfolio that is strongly cited by most players is suggestive of a pioneering or a blocking portfolio. In this case, the interactions seem to simply reflect the major applicants, marking the fact that the business has a limited size as the possibility for return on investments, which are high in the case of the introduction of novel radiopharmaceuticals.

### “Final Dataset”: Authors’ Classification and Analysis

A total of 221 patent documents, regularly granted and pertinent to technetium, were retrospectively found and analyzed to identify the major areas of discovery and developments in the last 20 years. Inventions with intellectual properties were collected with a wide geographic coverage, with a major focus on the largest worldwide markets (United States and European Union), including China, Japan, India, and other emerging countries. A more detailed chronological and geographical distribution of patent documents is reported and discussed later.

All these patent documents were confirmed to correlate with technetium-99m in the field of Nuclear Medicine by the authors and later further classified into specific groups uploading the Final Database in an Excel file. The file contained the following data for each patent: title, abstract, owner, inventor, and a link to the complete original document; the layout was then modified by adding a column in which the authors defined the classification and a column for a brief comment on the content. Furthermore, the data were analyzed in order to highlight time trends, country, classification, and object of the inventions. Time trends were intended as the evolution over time of patent data, considering both the priority year and the publication year. The first variable was assumed as the filing date of the first application.

The individual classifications were then collectively analyzed by all the authors in meetings scheduled in the following two months. After obtaining the consent of all authors to the classification, the various groups of patents were assigned to each author based on their specific field of interest. The result of every single evaluation of the contents of the patents of each group of documents was then shared among all the authors to obtain definitive consent.

The criteria used for defining boundaries were based on the current major lines of innovation, including novel modalities for supporting technetium-99m availability (*n* = 39 patents), labeling methods (*n* = 21), expanded use or reformulation of established radiopharmaceuticals (*n* = 19), new radiotracers (*n* = 65 inventions related to candidates with the ambition to become radiopharmaceutical and to approach the market), and molecular carrier which might be used as the building block for the design of new probes (*n* = 41).

A further final group, namely “other purpose of use”, was comprehensive of all inventions (*n* = 36) which were did not match the previous groups. In fact, this last group, which will not be discussed, includes patent documents that have as their object technological solutions concerning, for example, radiation protection or special waste treatment methodologies, rather than innovative technical solutions, and generator transport or elution devices and finally physical methodologies for measuring eluates.

### Alternative Processes for Implementing Technetium-99m Availability

The analysis of the patent documents dedicated to the technetium-99m production methods revealed the increase in the number of patent applications starting immediately after the deepest molybdenum-99 production crisis occurred in 2009–2010.^18^ This trend was evidently driven by the need for finding alternative solutions, whether they are aimed at the direct production of technetium-99m or indirect, involving the recovery of molybdenum-99.^18,44−48^ The interest in the most widely employed generator technology, characterized by several advantages, became particularly lively after 2016 when the period of serious molybdenum-99 reactor-based production crisis had already been overcome.

Indeed, among 39 examined patents related to the technetium-99m production processes about 60.5% concerned accelerator-driven direct or indirect production and 26.3% referred to generator-based production, while only 13.2% included ^99^Mo/^99m^Tc separation techniques.

Eleven patent documents were relevant to the development of alternative generator-based production of technetium-99m from the parent nuclide molybdenum-99. Among them, 7 regarded the development of purification methods or new purification chromatographic columns for the separation and recovery of technetium-99m from molybdenum-99 (JP2018038935, JP2004150977, and CN109701482 as examples), while 3 proposed new generator system configuration and devices (CN211636025U, CN211358389U, and CN208706260U). This cluster of patents was mainly concentrated in the 2016–2019 time period (only 2 patent documents were published in 2002 and 2012), consistent with the renewed interest in the optimization of generator technology. The technetium-99m generator had experienced many changes since it was introduced in the late 1950s, becoming a reliable technology supporting Nuclear Medicine practice for many decades with no significant need for further improvements over the years.^49^ In this context the most recent patent document (2019) analyzed concerned the separation of molybdenum-99 and uranium to be involved in the generator-based production method (WO2020188048).

A total of 13 patent documents were related to the indirect production of technetium-99m, mainly by photonuclear production of molybdenum-99 from a molybdenum-100 target (US20180061516, CN110544548, and WO2014/186898 as examples) and in one case by α irradiation of a zirconium-96 target (WO2006/028620). Among them, 5 patents were primarily focused on the extraction and purification of technetium-99m from photonuclear-produced molybdenum-99 with a low specific activity (e.g., WO2014/097269). This group of documents was filed in the 2011–2014 time frame, subsequent to the technetium-99m supply crisis. Only 3 of 13 patents were published in other years (2004 and 2019).

Another 7 patent documents were relevant to the direct production of technetium-99m, 5 of which addressed the production through the proton irradiation of a molybdenum-100 target and the development of the correlated technology (e.g., WO201192174, JP2021071435, and WO2013159201). One patent concerned the apparatus and methods for technetium-99m production by neutron-induced transmutation of molybdenum-98 (WO2013188793). Another document referred to a production cycle of both technetium-99m and technetium-94m through irradiation with charged particles of enriched molybdenum targets, comprising the recycling of the metal target (WO2012139220). This group of patent documents was again filed in 2010–2012 as an answer to a technological need. Only 2 of 7 documents were published more recently, 2019–2020, although most of the oldest patents were continuously updated during the following years.

Notable exceptions were represented by one document published in 2009, referring to both accelerator-based direct and indirect production of technetium-99m by the irradiation of a multilayer target of molybdenum-98 and molybdenum-100 (US9196388), 2 patents (2012–2013) describing muon-based production of technetium-99m (JP2014196997 and WO2014103712), and 4 patents (2017–2021) concerning different separation techniques of technetium from molybdenum, regardless of the type of production, based on distillation (WO201623112) or chromatography by using carbon fibers (CN111500861), adopting polyamide resin (CN106967882), or amino imidazole type ionic liquid loaded resin (CN110923480).

### New Labeling Methods

Technetium displays superior properties as a radionuclide for radiopharmaceutical development, not only for the ideal nuclear properties. The presence of multioxidation states and versatile chemistry promoted the discovery of several highly efficient and sophisticated labeling methods over the years, not comparable with any other radionuclide. The introduction of the ^99m^Tc-complex into a biologically active molecule is a fundamental part when designing a new agent and probably the most challenging, just relying on the skills of the radiochemist. Coordination studies have been conducted with the objective of facilitating and generalizing labeling for future cold-kit applications, stabilizing technetium-99m in a convenient building block while minimizing the possible effect on the targeting molecule. Not less importantly, new labeling procedures were implemented to meet high Specific Activities (SAs), a crucial requirement for the development of radiotracers targeting low-concentration substrates (i.e., receptors, enzymes, and other epitopes).^50^

**Figure 6:**
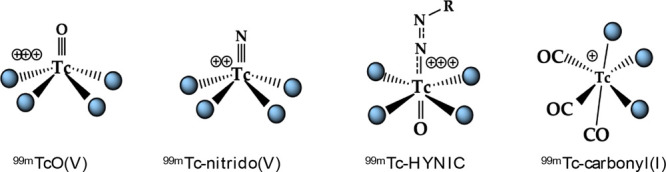
Recent ^99m^Tc-coordination models.

As a result of strong academic research efforts, several advanced chemical procedures were developed in the late 1990s and were available since 2000, based on the more recent coordination models over ^99m^TcO(V) and ^99m^Tc-nitrido(V) cores, besides the newest ^99m^Tc-HYNIC and ^99m^Tc-carbonyl(I) cores (Figure 6).

Despite the high efficacy of these approaches, they struggle to achieve widespread applications, probably due to the fact that just a few new ^99m^Tc-radiopharmaceuticals have been introduced in clinical practice. A couple of leading examples are ^99m^Tc-EDDA/HYNIC-Tyr3-octreotide, employed in the clinical setting for the detection of neuroendocrine tumors since 2005 (only recently obtaining MA in Europe with brand name Tektrotyd, Polatom, 2016) and ^99m^Tc-MIP1404 (trofolastat, Progenics, USA, in phase III clinical trials) as an imaging agent for prostate cancer.^51^

The observed group of 21 patents related to labeling processes was consistent with the strategic role of academic and research centers in the development of new stabilized ^99m^Tc-cores, alone (11) or forming a partnership with industries (8). The remaining 2 examined inventions belong to pharmaceutical companies; however, the patents were granted before 2005. The limited number of patent publications during the last years (7 patents were released after 2015) more likely reflects market stagnation and/or the reduced funding for dedicated research programs for innovation.

Among the examined methods, organometallic ^99m^Tc-carbonyl emerged as the most recurrent building block (7 patents, some examples are WO200125243, WO2018107526, WO2013172616), due to its great stability and the large variety of exploitable ligands. For these reasons, the novel [^99m^Tc(CO)_3_]^+^ core has been considered for a long time the ideal building block for new ^99m^Tc-target specific imaging agents, attracting investments from the radiopharmaceutical industry since the beginning.^52^ Unfortunately, after a first enthusiasm, this labeling method did not meet the market expectations and it is waiting to exploit its full potential. On the other hand, the oxidation state +V in the oxo or nitride form stabilized with various tetradentate ligands is a less recent but established and applied method. Following this approach, a few of the analyzed patents (6) related to the generalized labeling procedure for the technetium-99m incorporation into a large variety of targeting molecules which is suitable for kit formulation (for instance, WO2018109164 and WO200441311). Another 7 patents described nanoparticle labeling procedures or other highly specific labeling procedures (as in the case of WO2009129578 and WO201618896).

### Extension of Indication or Reformulation for Established Radiopharmaceuticals

The market size for radiopharmaceuticals is not comparable to other pharmaceutical branches for the restricted economic return. However, the Nuclear Medicine demand is expected to expand in the next decade, and in this area, SPECT agents still constitute the single largest product type in the global radiopharmaceuticals market.^53^

For these reasons, there might be an interest to consolidate this business and even to further optimize the existing products, which are often characterized by a short expiration data for lyophilized kits and the limited shelf life (hours) of the radiopharmaceutical after reconstitution. The latter is due not only to the intrinsic ^99m^Tc-decay but also to the molecular overall stability, which is hardly affected by the radioactivity present in the prepared injectable solution.^54^

A total of 19 analyzed patents disclosed inventions using authorized radiopharmaceuticals but with substantial differences in the indication of use or in the composition.

Radiopharmaceuticals usually approach market authorization with a single indication; however, the potential for multi-indications is often considered and typically emerges as a consequence of extensive clinical off-label use, more than a real drug repurposing concept. The off-label uses are strictly regulated by law; nevertheless if used within pertinent conditions this option might offer new challenges for Nuclear Medicine physicians as well as a potential benefit for patients. These opportunities might also be exploited to identify rare clinical indications, which are obviously less financially attractive for the pharmaceutical industry.

Among the examined patents, the innovation for the use of established radiopharmaceuticals was found in 10 documents, related to an extension of indications (4) for a ^99m^Tc-agent in clinical practice, or to an existent ^99m^Tc-agent respecting the indication and associated with a new nuclear imaging procedure, to a modality or multimodality, and to a method of acquisition or elaboration of the data, thus resulting in an improved imaging practice (6). A few significant examples are reported below, in order to give to the reader a better understanding. The recently approved agent ^99m^Tc-tilmanocept (Lymphoseek, Navidea Biopharmaceuticals Limited, USA and EU), based on a labeled mannosylated dextran construct, is indicated for lymphatic mapping and sentinel lymph node localization.^55^^99m^Tc-Tilmanocept is likely to replace ^99m^Tc-colloids and albumin macroaggregates, the standard diagnostic in the imaging procedure for the management of melanoma and early breast cancer. The potential clinical applications of this radiotracer, able to bind to CD206 on macrophages, might be also exploited for specific oncological or cardiovascular purposes as proposed in a couple of patents, for the detection and measurement of the concurrent inflammatory response^56^ (WO201918815).

^99m^Tc-TRODAT-1 (Global Medical solution, registered in Taiwan and mostly distributed in emerging countries) is a tropane derivative that has a high affinity for dopamine transporters (DAT) used in neuroimaging for differential diagnosis between Parkinson’s disease (PD) and nondegenerative tremors.^57^ One examined invention disclosed a multi-indication of this radiopharmaceutical, including a differentially diagnosing degenerative dementias, such as Alzheimer’s disease, Lewy Body, or frontotemporal dementias (US8491867). The method was based on tracer dynamic measurement and quantization immediately after administration and after a long period, as a detailed perfusion analysis. Another patent rather referred to a new imaging modality using two radiopharmaceuticals ^99m^Tc-TRODAT-1 and ^123^I-ADAM to be used in the same scan acquisition for the simultaneous detection of aberration on dopaminergic and serotoninergic systems.^58^

All these examples are representative of radiopharmaceuticals with limited and defined application fields. At present the ^99m^Tc-market is still paying more attention to some radiopharmaceuticals which generated a very high income during the past two decades, among them sestamibi (Cardiolite as brand name and the generics available from 2008 Stamicis, Adamibi, MibiSPECT, Technemibi), tetrofosmin (Myoview), and many bone agents (several functionalized diphosphonates).^59,60^ A total of 10 patents correlated to this class of radiopharmaceuticals, with lost patent protection, whereas all inventions disclosed new improved processes for the preparation of generics of the most widely employed radiopharmaceuticals.

Examining the intellectual properties, western pharmaceutical companies appeared less interested in building a market of generic radiopharmaceuticals, probably for less investor confidence about the future. Only two patents referred to a new composition for the preparation kit for ^99m^Tc-tetrofosmin (brand name Myoview, Amersham Healthcare, later GE Healthcare), which represented one of the latest blockbusters in the radiopharmaceutical SPECT-market and a prominent example of a patent family evolution for radiopharmaceutical kits.^61^ Tetrofosmin is extremely sensitive to atmospheric oxygen, making the manufacturing and handling of the kit complicated for industries, as well as for the final radiopharmaceutical preparation before clinical use in Nuclear Medicine units.^62^ Since 2000 new tetrofosmin formulations have followed one another with the aim to overcome these constraints, while Jubilant (US) and ROTOPmedipharma (EU) filed two patents approaching the generic market very recently.

On the contrary, the oriental market seemed to open to a reasonable prospect of future commercial success with several patents (*n* = 8) related to a new formulation for largely employed ^99m^Tc-radiopharmaceuticals, such as diphosphonates, ethyl-cysteinate dimer (ECD) and colloids (CN103203032, CN111920967, CN111973763). China was confirmed to be a key player as a main emerging market, including in the field of medical isotope-related technologies (4 patents). In recent years, the radiopharmaceutical industry has gradually attracted the attention of investors also thanks to specific government strategic plans, which provide a roadmap in driving forward the promotion of the research, development, production, and clinical application of radiopharmaceuticals in the country. China is expected to be one of the most important markets in the near future offering renewed opportunities for investors in Nuclear Medicine both in established and emerging technologies.^63^

### New Radiotracers

The development of new diagnostic agents follows two main preliminary steps: the identification of the biological process indicative of the disease to be investigated and the detection of the molecular target indicative of the pathologic process. This target will virtually represent the chemist’s workbench for the design of a new chemical structure, which is intended to be labeled with a radioisotope for a medical application. The considerable advances in molecular cell biology and the use of molecular modeling, as a potent tool for studying crystal structures, have boosted the development of small targeting molecules with optimized properties to specifically target receptors, enzymes, or other structures expressed on the cell surface or in the surrounding environment.^64^

In this scenario, radiochemistry maintained its crucial role in the synthesis of novel radiotracers, conducting fundamental research, striving for technological progress, as well as training talents and personnel in the field of radiopharmaceuticals. The development of a new radiotracer is not easy of course, involving specific expertise and technologies with limited availability.

However, the effort of national research centers, nuclear medicine laboratories, and, not least, academic and hospital radiopharmacies, is generally not able to economically support the complete approval pathway for a clinical use; therefore private companies have become active partners for Nuclear Medicine.

The radiotracer pipeline has a bottleneck shape, typical for conventional drugs, where only a few of the numerous candidates with suitable in vivo properties eventually become radiopharmaceuticals. In the case of radiopharmaceuticals, industries generally provide funding for further clinical trials only in the case of encouraging preclinical and clinical results (phase I and II), considering that a radiotracer entering phase III has a relatively high chance to reach the market. And this may not be enough for technetium-99m, since the competition generated by the emerging role of PET with fluorine-18 and gallium-68 radiopharmaceuticals should also be taken into account. As Zimmerman et al. discussed, new radiopharmaceutical acceptance by investors is ideally governed by the existence of a medical need, a favorable Health Technology Assessment outcome (HTA), and an addressable market, considering potential competitors not only in the field of nuclear technologies.^16^ This is consistent with a concise number of patents that might apply later for marketing authorization, a long and expensive process, which needs to be adequately attractive for the pharmaceutical industry.

A total of 65 patents were found to be correlated to new radiotracers, with the ambition to become ^99m^Tc-radiopharmaceuticals. Most of the examined patents disclosed inventions for oncological applications (36), clearly indicating the consolidated role of nuclear imaging procedures for supporting accurate management decisions and improve cancer patients’ outcomes. Since the beginning of 2000 inventions were filed homogeneously by western countries (USA and EU) and emerging countries (oriental countries such as Korea, China, Japan), as well as for intellectual properties of industry, academic, or research institutes. During the last 5 years, from 2015, a shift toward an oriental prevalence is notable (of a total of 12 patents, 8 patents from China versus 3 from the USA, 1 from Mexico, and none from the EU) consistent with the previously discussed evolving role of China in the field. Among the disclosed inventions some examples are a ^99m^Tc-labeled PD-L1 targeting peptide (CN111320675), with high specificity for the inhibitory checkpoint molecule PD-1, a HER2 specific ^99m^Tc-labeled peptide (WO201287908), ^99m^Tc-RGD (WO2011149250), and ^99m^Tc-neoantibodies (WO201275023).

**Figure 7:**
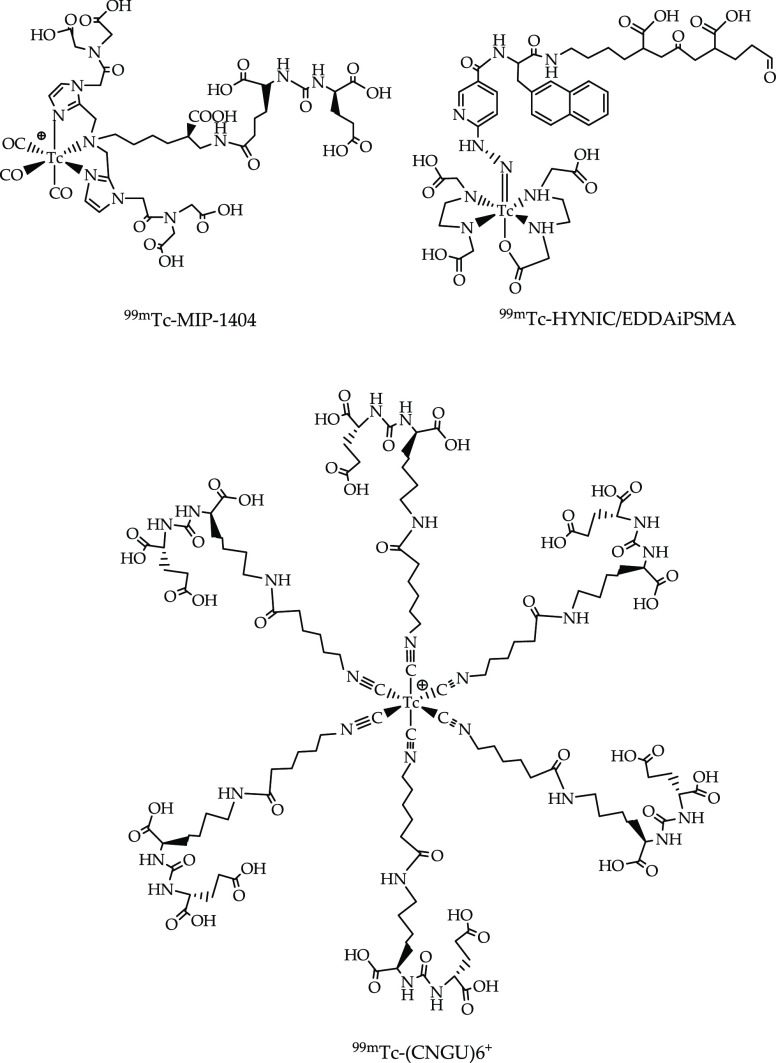
^99m^Tc-radiotracers for imaging prostate cancer.

An interesting case is related to a couple of inventions disclosing the development of radiotracers for imaging prostate cancer as alternatives to the previously cited ^99m^Tc-MIP1404 (WO200858192). In the proposed inventions, ^99m^Tc-HYNIC/EDDAiPSMA (WO2017222362) and [^99m^Tc-(CNGU)_6_]^+^ (CN112209970), technetium-99m is differently coordinated and functionalized to the same targeting molecule, which specifically binds prostate-specific membrane antigen (PSMA) (Figure 7). While the motif Glu-urea-Lys remains the same for all radiotracers, technetium-99m is stabilized into a [^99m^Tc(CO)_3_]^+^ core (^99m^Tc-MIP1404), a ^99m^Tc-HYNIC core (^99m^Tc-HYNIC/EDDAiPSMA), or a ^99m^Tc-isonitrile core for [^99m^Tc-(CNGU)_6_]^+^, thus resulting in substantially distinct radiotracers covered by different intellectual properties.

Radioguided surgery has been proposed as a method to improve intraoperative detection and clearance of metastatic lymph node involvement correlated to prostate cancer. Technetium-99m radiopharmaceuticals targeting PSMA are considered ideal candidates to use with this technology, that is, highly promising because of the large radioisotope availability and the established practice with gamma probes, and may improve oncological patients’ outcomes.^65^

Nanotechnology can also provide an excellent platform for the development of novel tracers and to support hybrid multimodality imaging systems.^66^ Since 2000, scientists have worked remarkably to translate nanomedicine into clinical practice with no exception for technetium-99m, trying to improve the performance of the existing radiopharmaceuticals or for new indications using inorganic and organic radiolabeled nanoparticles, as demonstrated by a number of filed patents. Multiple strategies have been developed to obtain highly stable and efficiently radiolabeled nanoparticles with ^99m^Tc. For example, ^99m^Tc-labeled liposomes and antimony trisulfide nanocolloids (US7264791) have been proposed, already in 2002 and 2004, as an alternative to classical radiopharmaceuticals for sentinel lymph nodes. A few years later, published patents referred to labeled organic germanium nanocolloids (US20070224117) and iron oxide (Fe_2_O_3_) nanoparticles (US20090035201) as new agents, respectively, for spleen imaging and for various diseases including tumors, contagious diseases, and genetic defects. During recent years more sophisticated organic macromolecules, such as functionalized dendrimers (WO201372071), gold nanoparticle binding peptides, AuNPs (US20140314668), and magnetic nanocrystals coupled to fluorophores (US20220031873), were patented as promising carriers upon which to load several imaging agents, including technetium-99m, to obtain multimodal imaging SPECT/CT or SPECT/MRI.

The remaining examined 27 patents disclosed radiotracers for cardiovascular and neurologic applications, for monitoring organ functionalities, such as for kidneys, lung or pancreas, or for other specific purposes.

### Molecular Carriers

The identification and validation of a target representative of a disease is the first requisite for the design of a new class of radiotracers, as mentioned before. The following key goal will be to find a way to deliver through suitable carrier molecules as much radioactivity as possible to the target for a prolonged period of time in order to obtain the desired diagnostic or therapeutic performances. Of course, ideal carriers incorporating radioisotopes should specifically bind the chosen target with negligible or absent interaction with healthy tissues.

Target identification is often performed by screening literature reports mainly based on autopsy and proteomic and genomic studies from academic and industrial researchers. Since Nuclear Medicine techniques are traditionally applied to a broad range of pathologies, the molecular targets to be explored for the development of suitable carriers are immense. On the other hand, the efficient and stable incorporation of a radioisotope into a carrier, preserving the affinity for the target, is not always possible. As a direct consequence, some carriers exhibit major flexibility, being potentially labeled with a wide range of radionuclides, while others are prone to be associated exclusively with one or few radioisotopes, sharing similar chemical properties.

As observed in the examined documents concerning the development of novel molecular carrier or carriers with potential in Nuclear Medicine, patent owners adopted the strategy to extend the combination of molecular vector with the largest range of radionuclides. This is also congruent with the intent to protect as much as possible the intellectual properties of the invention, with a cost-saving solution and, not second, preventing competitors from exploiting the business potential with slightly modified alternatives.

A relevant number of patent documents (*n* = 41) covered multiple inventions in a single patent, focused on a novel carrier or a class of carriers. Inventions described in the same patent were mainly represented by tracers based on the same carrier scaffold with small chemical modifications or different radioisotopes of choice, including technetium-99m among them. Few cases reported also carriers with multiple fields of application beyond the use in combination with radioisotopes. Their uses encompassed other imaging modalities or therapeutic purposes, thus overstepping the boundaries of an exclusive application in Nuclear Medicine.

Patent publication activity nearly reflects the progressive shift of innovation in Nuclear Medicine from SPECT to PET radiopharmaceuticals, as a consequence of the introduction of the first commercial PET-CT scanners in 2001^67^ and the emergency regarding technetium-99m availability later. However, once the crisis was over, technetium-99m also benefitted from the rise of a new age in Nuclear Medicine, thanks to its longstanding use and well-known chemistry.

In more detail, two examined patents disclosed inventions for the diagnosis of Alzheimer’s disease (WO200216333, WO200275318), while three patents by Bristol-Myers Squibb, filed between 2001 and 2006, disclosed compounds and methods for imaging myocardial perfusion and cardiac innervation and monitoring of various cardiovascular diseases confirming the Big Pharma interest in the early 2000s in the cardiovascular radiopharmaceutical market (WO200267761, WO200883056, WO200313346). More recently, with the exception of two patents disclosing methods of diagnosis of infection and inflammation, all the other inventions related to compounds and methods for oncological applications, confirming once again the role of technetium-99m for supporting accurate management decisions and improved cancer patient outcomes.

Among these, the most recent patents relate to inhibitors of CXC receptor 4 (CXCR4)–G protein-coupled receptor (GPCR) heteromers (CXCR4-GPCR heteromers) associated with cancers (WO2019124951); a somatostatin analogue (CN108586600); an rk-polypeptide radiopharmaceutical targeting HER2 (WO2020238795); PSMA binding ligand-linker conjugates, and methods for delivering therapeutic, diagnostic, and imaging agents (WO200926177).

Finally, from the analysis of the publications reported in the literature in the last two decades, it is curious to observe at a first glance that the trend of the studies carried out on gallium-68 radiopharmaceuticals for molecular targets is similar to the development of those containing technetium-99m. This corroborates the evidence of increased interest in a transversal approach of molecular carriers for all Nuclear Medicine modalities. Particularly, it seems that the surge in studies carried out on gallium-68 radiopharmaceuticals has unknowingly also boosted the development of technetium-99m tracers, suitable to be used in combination with the more disseminated SPECT instrumentation.

Indeed, the development of effective gallium-68 radiopharmaceuticals during the last years has helped to mitigate the general belief that the imaging sector of specific targets such as the receptor was a prerogative of PET radionuclides, such as fluorine-18 and carbon-11, considered as natural elements, with negligible impact on the biospecificity of labeled molecules.

### Patent Document Highlights

**Figure 8:**
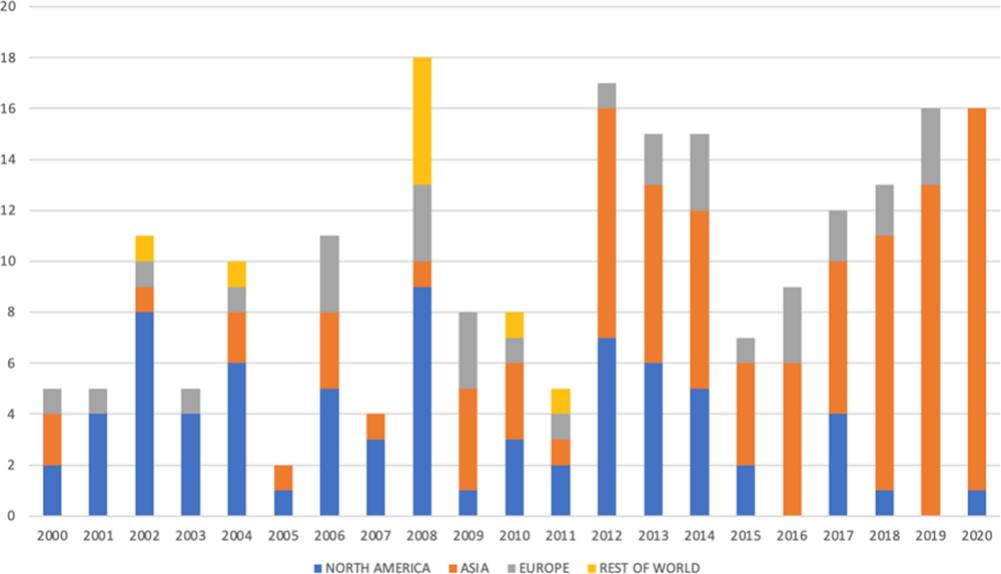
Geographical and chronological patent distribution worldwide of the final data set (*X*-axis reports geographical macroareas per year; *Y*-axis reports the number of patents).

A focus on the trends in the development of technetium-99m technologies is among the intents of this scientific contribution, identifying the areas of contraction of innovation or where significant advances have been made over the past two decades. Technetium-99m is one of the longest standing radionuclides with medical applications in a broad range of fields and probably the most prominent example. Its flexible attitude and wide availability have boosted the interest for the development of new diagnostic agents and supporting technologies since the discovery of ^99^Mo/^99m^Tc generators, and it is still promoting innovation in nuclear medicine until recent times. In the previous sections, illustrative examples from the extracted data of patents filed by industry and academic, research, or hospital institutions have been selected and presented, with an emphasis on the progress in technologies that trace the emerging lines of research. For a last comprehensive statistical analysis, the patents contained in the final data set were finally evaluated in the time period 2000–2020. The last two years considered in the initial data set were excluded for incomplete data, since filed patents were still partially confidential to the patent office (patent applications are automatically published after 18-months from their earliest priority date), as well as the patents with priority filing before 2000. The considered 212 patent documents were categorized per group (respecting the current major lines of innovation discussed before), year, and origin of applicants. The chronological and geographical distribution are indicated ichnographically in Figure 8.

At a first glance, the new refined analyzed data renders the same picture obtained classifying the patents in the final data set, where the radiopharmaceutical remains the ultimate goal of research deserving patent protection, with the highest number of patents filed, followed by the other groups defined by the chosen criteria.

However, when analyzing this data per year (Table 1), major patent activity outputs for lines of innovation were identified with remarkable observation mentioned below.

**Table 1:** Analytical Distribution of Patent Documents by Year and by Principal Category

year	new radiotracers	extension of indication or reformulation for established radiopharmaceu ticals	new labeling methods	alternative processes for implementing ^99m^Tc availability	molecular carriers
2000	1	0	1	0	3
2001	1	0	0	0	4
2002	3	0	4	1	3
2003	5	0	0	0	0
2004	3	1	2	1	2
2005	2	0	0	0	0
2006	2	2	1	0	2
2007	4	2	0	0	1
2008	6	1	2	0	0
2009	2	1	0	1	3
2010	6	0	0	3	0
2011	3	0	0	2	0
2012	2	2	1	6	4
2013	2	0	1	4	4
2014	3	1	2	4	3
2015	3	0	1	0	2
2016	1	1	2	3	2
2017	3	4	1	2	1
2018	4	2	1	3	2
2019	1	0	1	7	1
2020	8	2	0	2	0

**Figure 9:**
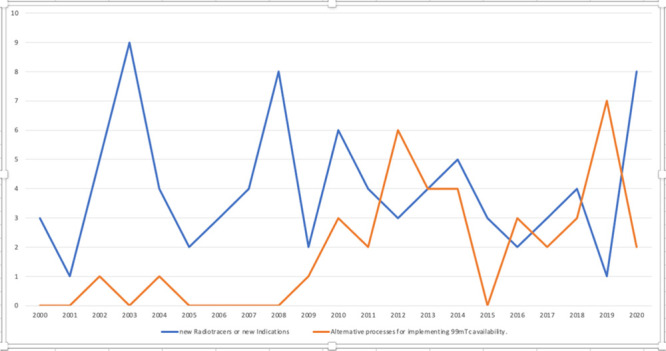
Patent documents trend of “New Radiotracers/new indications” and “Alternative processes ^99m^Tc availability”. *Y*-axis reports the patents number.

Radiopharmaceuticals, and correlated extension of indication/formulation, maintained high output, albeit discontinuously, during the first decade. This is likely due to the confidence to use technetium-99m novel radiopharmaceuticals or established radiopharmaceuticals for new indications in clinical practice. This positive trend declined for many years, just after the first major crisis of molybdenum availability in 2009–2010 for obvious reasons, to progressively and unexpectedly re-increase from 2017 until reaching intense activity in 2020 (Figure 9).

There are probably many reasons that might justify the rediscovery of interest in technetium-99m radiopharmaceuticals, determined by scientific, practical, and economic considerations. One of them could be the unparalleled impetus for developing new radiotracers, such as PSMA diagnostic agents, especially if associated with γ emitters for which instrumentation is highly available and overall costs are competitive with respect to PET examinations. Moreover, PSMA is nowadays the most important specific target to be studied for the broad implication into the clinic, but it is not unique. A number of new molecular carriers with favorable characteristics are already available for the development of new probes, not only for nuclear medicine applications. Last but not least the rising interest in the design of specific combinations of magnetic resonance and nuclear imaging contrast agents for multimodal assessments (e.g., PET/MRI) may contribute to the diffusion in clinical practice of new diagnostic and, in general, new molecular imaging approaches.^68,69^

Another potential reason is the great impulse given by the consolidation of alternative technologies for sustaining the production of technetium-99m. As reported in Figure 9, patent productivity for alternative processes for implementing technetium-99m remained almost silent for several years and then started as a response to the 2008–2009 and 2012–2013 global shortages, until becoming established in more recent years (and being the most representative in 2019). The nuclear medicine community is now more accustomed to deal with the not uncommon difficulties in the fragile technetium-99m supply chain; however arising technologies able to overcome such limitations help to mitigate some uncertainties about the future of technetium-99m, and thus create a renewed enthusiasm.

A last reason to consider is related to market movements and the needs of a modern and sustainable healthcare system, thus referring to the direct interest for the development and patent protection of an invention in a specific country.

When analyzing countries (Table 1, Figure 6) by their number of applicants (private or public institutions with residence in these countries), the USA and China stand out as expected, as the most productive nations with 73 and 56 filed patents, respectively, covering more than half of total analyzed patents. This observation was consistent when examining the distribution for world geographic areas, with the prevalence of the production in Asia (45%), thanks to the involvement of other dynamic countries such as Japan and Korea primarily. After the second output position of North America (35%), Europe originated the third-highest number of technetium-99m related inventions (16%), while the rest of the major areas in the word completed the overview with a minor contribution (4%).

Interestingly, not only is a general increase of patent activity observed in the second decade, but a shift in the hegemony in the field from the western to Asiatic countries is particularly evident starting from 2012. Considering that nearly half of the patents have been filed in the origin country of the applicants and thus where the applicant wants the invention to be protected, this information gives us an indication of which are considered the most important markets for investors in the technologies correlated with technetium-99m nowadays, while maintaining a general interest for disseminating the innovation in all the developed countries.

## Conclusion

There are many uncertainties as to what will be the role of technetium-99m in the near future in the rising PET technologies combined with therapeutic agents. The frequent difficulties concerning this radioisotope supply and the great interest in new radiopharmaceuticals directed toward specific targets (i.e., PSMA) legitimate the questions regarding the need for new tracers labeled with ^99m^Tc and its under-attack supremacy in Nuclear Medicine.

We tried to answer these questions with an assessment of patent data related to technetium-99m filed during the last 20 years, including patent count and analysis of supplementary information extracted from the documents.

Patent analyses provide information on technological innovation and the use of patent search engines, free or paid platforms based on patent databases, demonstrated to be a simple and immediate source to measure innovation performance for both academic and industrial researchers. These powerful tools are able to generate data collection containing patent documents, toward validation and cleaning processes, addressing the required information and identifying trends and topics of interest. However, while dedicated software is essential for handling a number of documents, data cleaning might lead to imprecise results mostly due to an evident patent heterogeneity. Indeed, a further human refining process is required in our experience, highlighting some limitations of these platforms, especially in the management of poor-quality documents.

Focusing on the actual situation and future horizon, the place of SPECT imaging using technetium-99m radiopharmaceuticals is still solid. This long-standing workhorse in Nuclear Medicine will remain essential for the specialty in the next years, thanks to a large variety of available radiopharmaceuticals and their low costs and wide accessibility compared with PET agents.

However, the past problems for ^99m^Tc-availability and the aggressive competition by PET technologies irreversibly destabilized its long-lasting hegemony. The general overview is controversial: patent applications, especially in the eastern economies, such as China and other emerging markets, are increasing, while those of western developed countries are stagnating, with some exceptions for the United States, a traditional cradle of innovation.

Patent count and analysis described the state of health of technetium-99m in Nuclear Medicine also giving a picture of new trends and likely anticipating new adoptions into the clinical practice. However, it is hard to predict if the most recently developed tracers will be well-accepted as new agents in clinical routine since very few ^99m^Tc-radiopharmaceuticals emerged over the last two decades. This is not a problem of chemistry or biology, but rather due to the rising requirements imposed by the market, companies, and Regulatory Authorities. Furthermore, other factors might play a role in changing diagnostic requests linked to the procedures using technetium-99m, such as reimbursement rate policies or the increasing wealth in emerging economies. The evaluation of all these factors and their long-term effects is crucial for correctly addressing the future demand for technetium-99m radiopharmaceuticals.

In any case, even if market introduction is not realized as th eultimate objective, radiopharmaceutical research, especially for technetium-99m and fluorine-18, remains strategically important for the improvement of Nuclear Medicine, justifying efforts (success and defeat stories) at academic and industrial levels.

## Experimental Section

The ORBIT Intelligence system from QUESTEL (France) was used for the collection of technetium inventions disclosed in patents and patent applications (collectively named patent documents). The databases selected for patent search and retrieval were all ORBIT system patent databases, representing more than 96 countries.

The time period considered was patent documents having a priority date after January 1, 2000, and published until February, 14, 2022. Patent applications benefit from a secrecy period of 18 months after the first filing; therefore applications filed before February, 14, 2022, but still in the secrecy period are not considered in the present study.

The design of search strategies, according to the ORBIT Intelligence system syntax, was based on the construction of logical expression or search query. In a first step, the following logical expressions were searched in the claims: “+99MTC+” or “TECHNETIUM”. The extracted documents were grouped into 35 technology fields based on the IPC codes (International Patent Classification, http://www.wipo.int/classifications/ipc/en/) to exclude technology fields out of the scope of the present analysis. Patent documents belonging to the following IPC codes were excluded: human necessities, agriculture (A01P); electricity (H03#, H01L, H01M, H04#); textiles and paper (D02#, D21#, D21#, D03#); performing operations and transporting (B22C, B31#, B41#, B60#, B61#, B62#, B63B, B63C, B63G, B63H, B63J, B64#); physics (G08#, G02#, G07#, G06#, G11C, G10L, G21C); fixed constructions (E01#, E02#, E03#, E04#, E05#, E06#, E21#, E99#). After this processing, several patents and patent documents not pertinent to technetium-99m in life sciences were removed from the cluster, originating a first extracted data set (First Data set).

This result was further refined using ORBIT Intelligence by extracting the patent documents comprising the terms “+99MTC+” or “TECHNETIUM” in the independent claims only first, and second confirming the presence of the keyword “RADIO+” anywhere in the title, abstract, description, and claims fields. The obtained pool of patents (Intermediate Data set) was then filtered for the legal status “alive”, excluding all the expired documents, thus obtaining a Final Raw Data set.

This series of patent documents were finally independently analyzed by the group of authors. The authors independently reviewed each patent, confirming correct inclusion (or reporting the need for exclusion) after cleaning operations. Disagreements were examined and resolved by consensus.

The remaining records, named Final Data set, were manually scrutinized by the authors in order to classify the claims comprising each patent into six representative categories, coded “New labelling methods” (referring to novel technetium-99m core labeling modalities), “Alternative processes for implementing technetium-99m availability” (describing new processes for the ^99m^Tc-production), “Extension of indication or reformulation for established radiopharmaceuticals” (dedicated to ^99m^Tc-radiopharmaceuticals already in the market with modified formulations or addressing other diagnostic questions), “Radiotracers” (describing new radiopharmaceutical candidates), and “vectors” (focusing on molecular carriers which might be labeled with technetium-99m). An additional group, “Other purposes” collected documents which did not match the previous groups.
